# Daytime skin temperature and nocturia in the subsequent night among community-dwelling older adults in the cold season in the HEIJO-KYO study

**DOI:** 10.1186/s40101-026-00442-w

**Published:** 2026-08-04

**Authors:** Daisuke Tamaru, Kenji Obayashi, Yoshiaki Tai, Yuki Yamagami, Kazuki Okumura, Hiromitsu Negoro, Keigo Saeki

**Affiliations:** 1 Department of Epidemiology, Nara Medical University School of Medicine, 840 Shijocho, Kashihara, Nara 634-8521 Japan; 2 Department of Psychiatry, Nara Medical University School of Medicine, 840 Shijocho, Kashihara, Nara 634-8521 Japan; 3 Department of Urology, University of Tsukuba, 1-1-1 Tennodai, Tsukuba, Ibaraki 305-8577 Japan

**Keywords:** Nocturia, Skin Temperature, Cold temperature, Body Temperature Regulation, Environment and Public Health

## Abstract

**Background:**

Both Nocturia and cold exposure are associated with higher mortality and other health problems. The previous studies already indicated the significant association between nocturia and air temperature. Skin temperature might be superior to air temperature in that it can show the individualized impact of cold exposure. The current study aimed to investigate the association between distal–proximal skin temperature gradient (DPG) and nocturia among older adults in the living environment.

**Methods:**

The participants of this cross-sectional study were 558 community-dwelling people (≥ 60 years old) in Nara, Japan. Distal (wrist and ankle) and proximal (abdomen) skin temperatures were measured every minute for 48 h starting at noon in the cold season. DPG was calculated as distal skin temperature minus the proximal one. Then, the prevalence of nocturia (≥ 2 voids per night) was evaluated based on the standardized diary. We focused on the prevalence of nocturia on the second night and DPG of the daytime just before. We constructed logistic regression models to calculate the odds ratio for nocturia associated with a 1 °C increase in daytime DPG. Furthermore, we also analyzed the association between nocturia and nighttime DPG.

**Results:**

The means (standard deviation) of daytime distal–proximal skin temperature gradient among participants with and without nocturia were − 3.41 °C (1.83) and − 2.93 °C (1.63), respectively (*P* = 0.004). Further, a 1 °C higher daytime DPG was significantly associated with lower odds for nocturia (crude odds ratio: 0.85; 95% confidence interval: 0.76–0.94; *P* = 0.003). The association was independent of potential confounders (adjusted odds ratio: 0.79; 95% confidence interval: 0.69–0.90; *P* < 0.001). The potential confounders included basic characteristics (age, gender, body mass index, current smoking, and ethanol intake), clinical variables and medications (blood pressure, estimated glomerular filtration rate, depressive symptoms, calcium channel blockers, diabetic medicines, diuretics, and sleeping pills), and socioeconomic status (education ≥ 13 years and high household income). Nighttime DPG and nocturia showed the similar association (adjusted odds ratio: 0.65; 95% confidence interval: 0.48 − 0.88; *P* = 0.005).

**Conclusions:**

A higher daytime DPG in the living environment was significantly associated with lower odds of nocturia in older adults.

## Introduction

The prevalence of nocturia (≥ 2 voids per night) in people who are in their 60 s is about 30%, and it is higher in people who are in their 70 s (> 40%) and 80 s (> 50%) [1]. Patients with nocturia had a 30% higher all-cause mortality than those without it [2]. Although the causations are not completely clear, nocturia may partly contribute to health outcomes. Nocturia is associated with a lower quality of life [3, 4], mental well-being [3, 5], and work productivity [3], and a higher prevalence of diabetes [4, 6], hypertension [7], cardiac diseases [4, 6], stroke [6], obesity [4], and sleep disorder [6] in cross-sectional studies and with a higher fracture risk [8] in a longitudinal study. Cold exposure is associated with a higher all-cause mortality [9] and incidence of cardiovascular diseases [10] and nocturia in both physiological [11–13] and epidemiological studies [14–16].

Previous studies have reported a significant association between nocturia and lower outdoor [15] and indoor temperatures [14]. Cold-induced diuresis is considered a physiological response that may help counteract cold-induced increases in central blood volume and blood pressure. Cold exposure induces peripheral vasoconstriction, which may increase venous return and urine production [17]. The influence of cold exposure may be modified by other factors such as adiposity, clothing, sensitivity to cold exposure, and thermogenesis. Therefore, skin temperature can be superior to outdoor and indoor temperatures to quantify and exactly assess the effect of cold exposure. Distal skin temperature is more easily affected by environmental temperature. On the other hand, proximal skin temperature is less affected by environmental temperature and has slight fluctuations. Distal–proximal skin temperature gradient (DPG; distal skin temperature minus proximal skin temperature) has been sometimes used as an indicator for estimating blood flow in distal skin regions, which depends on degree of vasodilation [18]. To our knowledge, there has not been a study about skin temperature in the living environment and nocturia. The current study aimed to investigate the association between daytime DPG and nocturia in community-dwelling older adults in the cold season.

## Methods

### Study design and protocol

This cross-sectional study was conducted as a part of the baseline survey in the Housing Environments and Health Investigation among Japanese Older People in Nara, Kansai Region (HEIJO-KYO) study, our community-based cohort research targeting people 60-years-old and older [19]. We measured skin temperature and indoor temperatures for 48 h, starting at noon in the cold season (October–April) and including two nights. The participants recorded the number of nocturnal voids on the second night, rising time, and bedtime in standardized diary. We instructed the participants not to drink alcohol or smoke during the measurement. The association between daytime DPG and the prevalence of nocturia on the following night was investigated. We conducted the current study in accordance with the Declaration of Helsinki, and the study protocol was approved by the Nara Medical University Ethics Committee (No. 301). All participants provided a written informed consent.

### Participants

Of 3,012 participants in the HEIJO-KYO study, all participants recruited between 2012 and 2014 (n = 589) received skin temperature measurements for 48 h in the cold season. After excluding 31 participants due to missing data on daytime skin temperature or prevalence of nocturia or bedtime, 558 participants were analyzed. Only when we used nighttime skin temperature in the analyses, we excluded four more participants due to missing values.

### Distal–proximal skin temperature gradient (DPG)

We evaluated skin temperatures of the wrist, ankle, and abdomen at one-minute intervals using Thermochron iButton DS1992L (Maxim Integrated, Dallas, TX) for 48 h. The sensors were attached to the dominant wrist’s flexor surface and the ankle’s lateral surface with a silicone band. In the right upper quadrant of the abdominal skin, the sensor was attached with Tegaderm transparent dressing (3 M, St. Paul, MN). Those measurement sites for distal skin temperature, wrist and ankle, were the most peripheral sites which enabled measurements under real-life conditions. A previous study reported the validity of skin temperature using iButton, and there have been a study measuring skin temperature at wrist [20, 21].

Distal skin temperature was the mean of the wrist and ankle skin temperature. DPG was defined as distal skin temperature minus abdominal (proximal) skin temperature. Daytime was defined as rising time to bedtime and nighttime as bedtime to rising time. In the current study, daytime skin temperature between the first and second night was used for analysis. We excluded data on wrist and ankle skin temperature below 20 °C and abdominal skin temperature below 25 °C. These outliers were below the mean minus four times the standard deviation (SD). The proportion of excluded data was < 0.2% of all skin temperature measurements (1,579,764). When we calculated the value of DPG, we used the average skin temperature in each participant.

### Nocturnal void and urine volume

Based on the diary, the prevalence of nocturia (≥ 2 voids per night) on the second night was determined. The participants stored the nocturnal and the first-morning urine, and the total amount of urine (total urine volume) was then evaluated. Single-voided urine volume was calculated as (total urine volume [mL])/(number of nocturnal voids + 1) [22, 23].

### Other measurements

The following demographic and clinical characteristics were investigated using a standardized questionnaire: age, gender, smoking, drinking, duration of education, household income, depressive symptoms (15-item Geriatric Depression Scale score of > 5), history of benign prostatic hyperplasia, and medication use. The prevalence of diabetes mellitus was determined based on medication use. Height and body weight were measured, and body mass index (BMI: kg/m^2^) was calculated. The estimated glomerular filtration rate (eGFR) was calculated with the formula: eGFR (ml/min/1.73 m^2^) = 194 × [serum creatinine (mg/dl)]^−1.094^ × [age (years)]^−0.287^. The result was multiplied by a correction factor of 0.739 for women. This is specified in the Evidence-Based Clinical Practice Guideline for Chronic Kidney Disease 2018 published by the Japanese Society of Nephrology [24]. We measured ambulatory blood pressure every 30 min with the monitor (TM-2430; A&M Co. Ltd., Tokyo, Japan) attached to the nondominant arms. Then, the mean was calculated as nighttime blood pressure. Actigraphy (Actiwatch 2; Respironics Inc., Murrysville, PA, USA) was attached to the nondominant wrists to measure sleep efficiency, which is the percentage of time from bedtime until rising time with physical activity below 40 counts/min. The living room and bedroom temperatures were measured at 60 cm from the floor every 10 min with iButtons. The outdoor temperature at 10-min intervals was obtained from the meteorological office in Nara (34°N). We asked participants to record the time when they got out of and into their houses, when they entered and exited bathroom, and when they went to bedroom in the standardized diary. We also calculated the percentage of bathing time relative to daytime. We calculated the average temperature to which each participant was exposed and defined it as the ambient temperature. To calculate ambient temperature, we used outdoor temperature, living room temperature, and bedroom temperature. We considered that the participants had spent their time at home in the living room, rather than in the bedroom.

### Statistical methods

Continuous variables with a normal distribution were presented as mean and SD. Dichotomous variables were expressed as numbers and proportions. Continuous and dichotomous variables were compared between groups using the Welch’s *t*-test and chi-square test, respectively. Using a generalized additive model, we estimated the fluctuation of the mean and 99% confidence interval of DPG according to the prevalence of nocturia.

We constructed logistic regression models to calculate the odds ratio (OR) for nocturia associated with a 1 °C increase in daytime DPG. *P* values were estimated using the Wald’s test. To calculate the adjusted OR, the multivariable logistic regression models included the following potential confounders: age > 70 years, gender, BMI of ≥ 25 kg/m^2^, current smoking, ethanol intake of ≥ 30 g/day, nighttime systolic blood pressure, eGFR, depressive symptoms, use of calcium channel blockers, diabetic medications, diuretics, and sleeping pills, education of ≥ 13 years, household income of ≥ 4 million JPY per year, and daytime ambient temperature. The similar analyses were conducted for nighttime DPG. Next, we imputed the mean values for the missing values of potential confounders.

Further adjustments were made for potential mediators such as sleep efficiency, time in bed, and single-voided and total urine volume in the nighttime. To assess whether gender modified the association between daytime DPG and nocturia, we made the multivariable model once more, which included interaction term. Due to multicollinearity, single-voided and total urine volume in the nighttime were not included in the same model.

In the stratified analysis, we divided participants into the quartile groups of daytime DPG according to gender, referred to as Q1–Q4 groups from the lowest to the highest. The logistic regression models were constructed to calculate the ORs for nocturia in each quartile group (referred as Q1 group). The basic characteristics, as shown in Table 1 (for male participants, plus benign prostatic hyperplasia), were adjusted. The association between daytime DPG and both total and single-voided urine volumes in the nighttime was analyzed using the linear regression model. To estimate the population-attributable fraction, the ORs of nocturia in Q1–Q3 groups with reference to Q4 group were calculated. All analyses were conducted using R version 4.0.3. ‘mgcv’ was used to establish generalized additive models.

## Results

Of 558 participants, 264 (47.3%) were men, and the median age was 70.0 (interquartile range: 11.8) years. The prevalence of nocturia was 29.6% (n = 165). Participants with nocturia had a significantly higher proportion of older (> 70 years) and male ones. Prevalence of nocturia was significantly associated with higher systolic blood pressure in the nighttime, eGFR, total urine volume, depressive symptoms, prostatic hyperplasia, longer time in bed, lower sleep efficiency, and lower single-voided urine volume in the nighttime (Table 1). Mean bathing time of the participants was 27.7 min, and it corresponded to 2.8% of the daytime. The numbers of missing values were as follows: 12 (2.2%) of “nighttime systolic blood pressure”, 4 (0.72%) of “depressive symptoms”, 1 (0.18%) of “benign prostatic hyperplasia”, 1 (0.18%) of “sleeping pills”, 1 (0.18%) of “calcium channel blockers”, 46 (8.2%) of “high household income”, 8 (1.4%) of “indoor temperature”, and 8 (1.4%) of “ambient temperature”.

**Table 1:** Characteristics of 558 participants by the presence of nocturia

	Nocturia	No nocturia	
	n = 165	n = 393	***p***
Basic characteristics			
Age > 70 years, n (%)	108 (65.5)	165 (42.0)	< 0.001
Male, n (%)	90 (54.5)	174 (44.3)	0.027
BMI ≥ 25 kg/m^2^, n (%)	46 (27.9)	119 (30.3)	0.571
Current smoker, n (%)	11 (6.7)	21 (5.3)	0.540
Ethanol intake ≥ 30 g/day, n (%)	29 (17.6)	63 (16.0)	0.654
Clinical variables			
nighttime SBP (mmHg), mean (SD)	119.7 (17.7)	112.0 (17.4)	< 0.001
eGFR (mL/min/1.73m^2^), mean (SD)	70.6 (16.1)	73.8 (15.1)	0.028
Diabetes Mellitus, n (%) ^a^	13 (7.9)	29 (7.4)	0.838
Depressive symptoms, n (%) ^b^	51 (31.1)	61 (15.6)	< 0.001
Benign prostatic hyperplasia, n (%) ^c^	26 (29.2)	26 (14.9)	0.006
Medications, n (%)			
Calcium channel blockers	55 (33.5)	103 (26.2)	0.080
Diuretics ^d^	11 (6.7)	18 (4.6)	0.311
Sleeping pills	20 (12.2)	36 (9.2)	0.278
Socioeconomic status, n (%)			
Education ≥ 13 years	41 (24.9)	123 (31.3)	0.127
High household income ^e^	55 (37.9)	158 (43.1)	0.290
Daytime air temperatures (°C), mean (SD)			
Outdoor temperature	7.78 (4.43)	8.02 (4.48)	0.567
Indoor temperature ^f^	14.83 (4.12)	15.53 (3.70)	0.063
Ambient temperature ^g^	13.50 (3.98)	13.88 (4.01)	0.307
Sleep parameters, mean (SD)			
Sleep efficiency (%)	81.0 (9.02)	85.6 (7.32)	< 0.001
Time in bed (min)	533.7 (83.5)	480.6 (77.7)	< 0.001
Nighttime urination, mean (SD)			
Total urine volume (mL) ^h^	740.6 (303.4)	519.5 (196.1)	< 0.001
Single voided urine volume (mL) ^i^	223.1 (92.5)	342.8 (134.4)	< 0.001

*SD* standard deviation, *BMI* body mass index, *SBP* systolic blood pressure, *eGFR* estimated glomerular filtration rate

*P*-values of continuous and dichotomous variables were determined with Welch’s t-test and Chi-squared test, respectively

^a ^Self-reporting of diabetic medication

^b^ 15-item Geriatric depression scale ≥ 5

^c^ Male participants only

^d^ Thiazide and loop diuretics

^e^ ≥ 4 million JPY per year

^f^ Mean of living room and bedroom temperatures weighted by staying time

^g^ Mean of outside and room temperatures weighted by staying time

^h^ Total amount of nocturnal and morning first urine volume

^i^ Total urine volume divided by the number of nocturnal voids plus 1

**Fig. 1:**
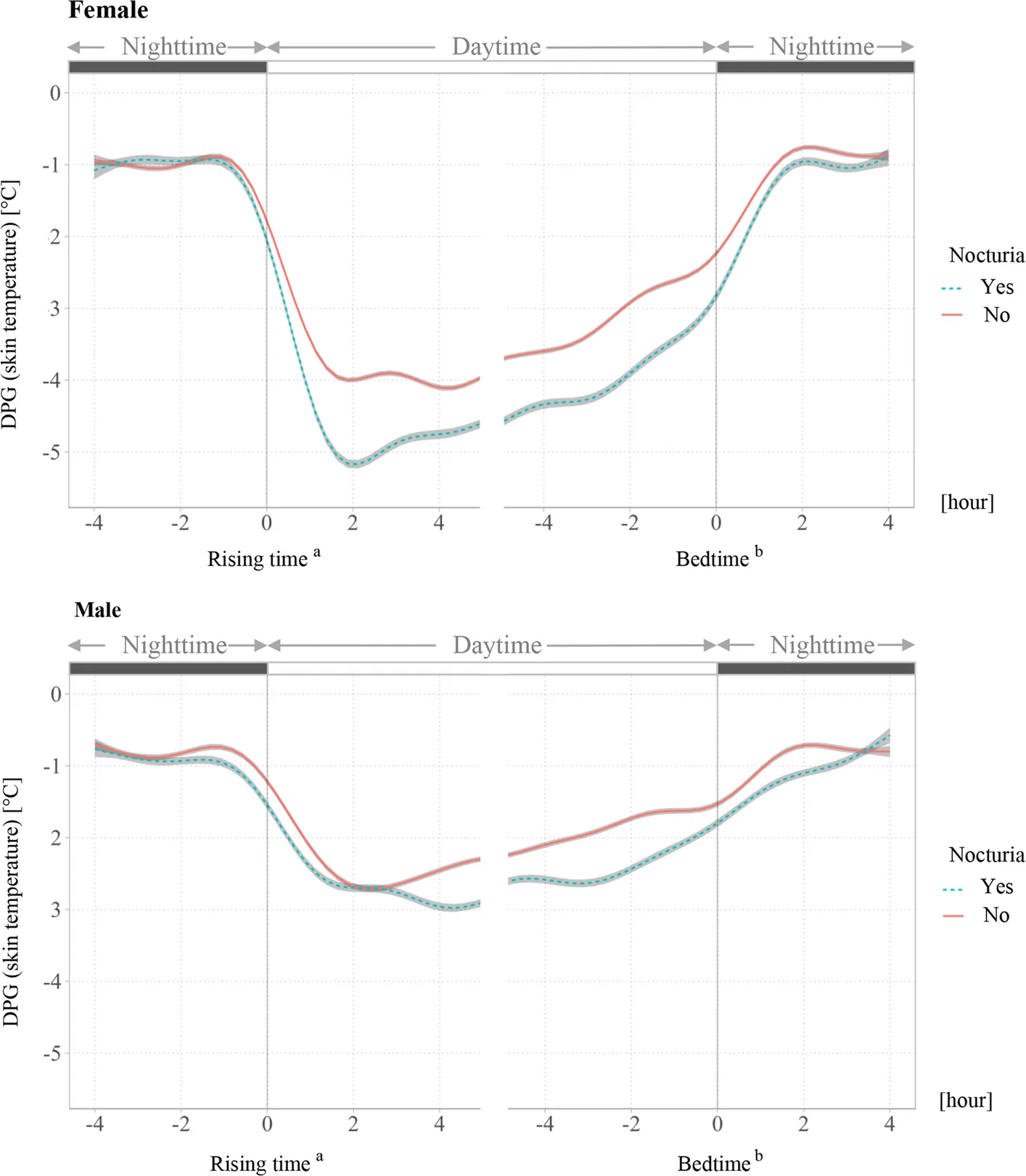
Gender-specific time-dependent change in DPG between participants with and without nocturia. DPG, distal–proximal skin temperature gradient: distal skin temperature (mean of wrist and ankle skin temperatures) minus abdominal skin temperature. The gray areas behind each line indicate 99% confidence intervals. ^a^ the time when each participant got out of their bed. ^b^ the time when each participant got into their bed. Daytime DPG in female was lower than in male. In each gender, those who had nocturia were likely to have lower daytime DPG

Participants without nocturia had a significantly higher daytime DPG (mean [SD]: − 2.93˚C [1.63] vs. − 3.41˚C [1.83], *P* = 0.004) and nighttime DPG (− 0.94˚C [0.65] vs. − 1.10˚C [0.66], *P* = 0.011) than those with nocturia (Table 2). Male participants had a higher daytime DPG than female participants (mean [SD]: − 2.33˚C [1.55] vs. − 3.75˚C [1.55], *P* < 0.001). Male and female participants with nocturia almost always had a lower daytime DPG than those without nocturia (Fig. 1).

**Table 2:** Daytime skin temperatures of 558 participants by the presence of nocturia

	**Nocturia**	**No nocturia**	***p***
Daytime skin temperatures (°C), mean (SD)	**n = 165**	**n = 393**	
Wrist	31.49 (1.54)	31.66 (1.53)	0.248
Ankle	31.34 (1.62)	31.51 (1.53)	0.236
Abdomen	34.82 (1.35)	34.52 (1.24)	0.012
Distal ^a^	31.41 (1.27)	31.58 (1.24)	0.147
DPG ^b^	−3.41 (1.83)	−2.93 (1.63)	0.004
Nighttime skin temperatures (°C), mean (SD)	**n = 164**	**n = 390**	
Wrist	34.59 (0.82)	34.87 (0.89)	< 0.001
Ankle	34.73 (1.09)	34.72 (1.00)	0.967
Abdomen	35.76 (0.50)	35.74 (0.49)	0.639
Distal ^a^	34.66 (0.68)	34.79 (0.71)	0.039
DPG ^b^	−1.10 (0.66)	−0.94 (0.65)	0.011

*SD* standard deviation, *DPG* distal–proximal skin temperature gradient

*P*-values were determined with Welch’s t-test

^a^ mean of wrist and ankle skin temperatures

^b^ distal skin temperature minus abdominal skin temperature

A 1 °C higher daytime DPG was associated with significantly lower crude odds for nocturia (OR: 0.85; 95% confidence interval [CI]: 0.76 − 0.94; *P* = 0.003). This association was consistent even after adjusting for potential confounders: basic characteristics (> 70 years of age, gender, BMI of ≥ 25 kg/m^2^, current smoking, and ethanol intake of ≥ 30 g/day), clinical variables (nighttime systolic blood pressure, eGFR, and prevalence of diabetes mellitus and depressive symptoms), use of medications (calcium channel blockers, diuretics, and sleeping pills), and socioeconomic status (education of ≥ 13 years and high household income) (model 3 – adjusted OR: 0.79; 95% CI: 0.69 − 0.90; *P* < 0.001) (Table 3).

**Table 3:** Odds ratios for nocturia associated with 1 °C increase in daytime distal–proximal skin temperature gradient (DPG)

	OR (95% CI)	***p***
Crude model	0.85 (0.76, 0.94)	0.003
Adjusted model 1 ^a^	0.80 (0.70, 0.90)	< 0.001
Adjusted model 2 ^b^	0.79 (0.70, 0.90)	< 0.001
Adjusted model 3 ^c^	0.79 (0.69, 0.90)	< 0.001
Adjusted model 4 ^d^	0.80 (0.70, 0.92)	0.002

*OR* odds ratio, *CI* confidence interval, *BMI* body mass index, *SBP* systolic blood pressure, *eGFR* estimated glomerular filtration rate

^a^ Adjusted for basic characteristics (age > 70 years, gender, BMI ≥ 25 kg/m^2^, current smoking, and ethanol intake ≥ 30 g/day)

^b^ Adjusted for variables in model 1 and clinical variables and medications (nighttime SBP, eGFR, depressive symptoms, calcium channel blockers, diabetic medicines, diuretics, and sleeping pills)

^c^ Adjusted for variables in model 2 and socioeconomic status (education ≥ 13 years and high household income)

^d^ Adjusted for variables in model 3 and daytime ambient temperature

The analyses for nighttime DPG showed that 1 °C higher nighttime DPG was associated with significantly lower crude odds for nocturia (OR: 0.70; 95% CI: 0.53 − 0.92; *P* = 0.012). This association was consistent after adjusting for the same potential confounders (model 7 – adjusted OR: 0.65; 95% CI: 0.48 − 0.88; *P* = 0.005) (Table 4).

**Table 4:** Odds ratios for nocturia associated with 1 °C increase in nighttime distal–proximal skin temperature gradient (DPG)

	**OR (95% CI)**	***p***
Crude model	0.70 (0.53, 0.92)	0.012
Adjusted model 5 ^a^	0.66 (0.50, 0.89)	0.005
Adjusted model 6 ^b^	0.65 (0.48, 0.88)	0.005
Adjusted model 7 ^c^	0.65 (0.48, 0.88)	0.005

*OR* odds ratio, *CI* confidence interval, *BMI* body mass index, *SBP* systolic blood pressure, *eGFR* estimated glomerular filtration rate

^a^ Adjusted for basic characteristics (age > 70 years, gender, BMI ≥ 25 kg/m^2^, current smoking, and ethanol intake ≥ 30 g/day)

^b^ Adjusted for variables in model 1 and clinical variables and medications (nighttime SBP, eGFR, depressive symptoms, calcium channel blockers, diabetic medicines, diuretics, and sleeping pills)

^c^ Adjusted for variables in model 2 and socioeconomic status (education ≥ 13 years and high household income)

After further adjusting for ambient temperature, the association between daytime DPG and nocturia remained significant (model 4 – adjusted OR: 0.80; 95% CI: 0.70–0.92; *P* = 0.002). The result was consistent in model 5 (further adjusted for sleep efficiency and time in bed, adjusted OR: 0.80, 95% CI: 0.69 − 0.92, *P* = 0.002), model 6 (further adjusted for nighttime single-voided urine volume, adjusted OR: 0.80, 95% CI: 0.69 − 0.93, *P* = 0.003), and model 7 (further adjusted for nighttime total urine volume, adjusted OR: 0.82, 95% CI: 0.71 − 0.95, *P* = 0.010). The interaction between daytime DPG and gender was not significant in model 3 (*P* = 0.23).

**Fig. 2:**
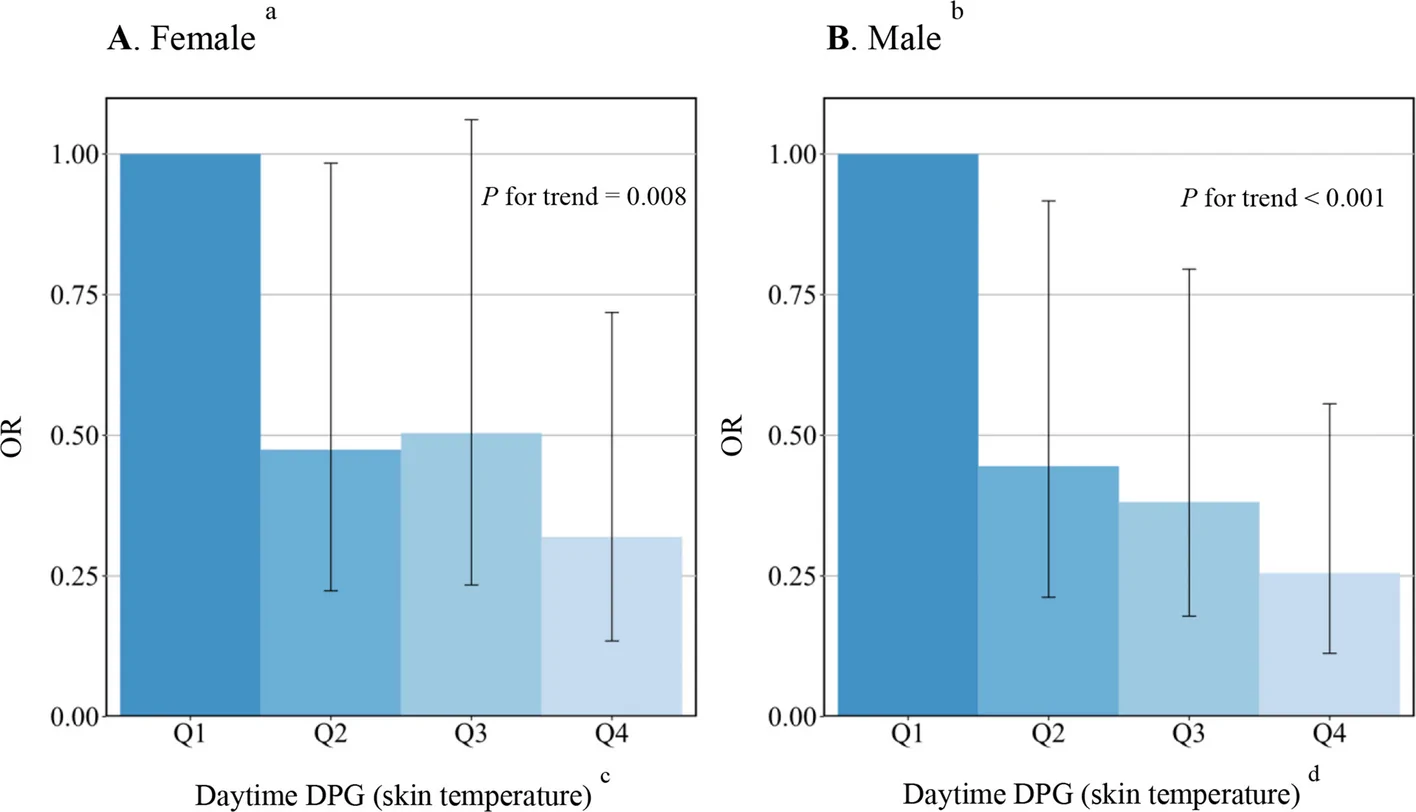
Adjusted odds ratios for nocturia by quartile groups of daytime DPG among males and females. OR, odds ratio; DPG, distal–proximal skin temperature gradient. Error bars present 95% confidence interval. ^a^ Adjusted for age > 70 years, BMI ≥ 25 kg/m^2^, current smoking, and ethanol intake ≥ 30 g/day. ^b^ Adjusted for benign prostatic hyperplasia in addition to variables in female adjusted model. ^c^ The ranges of DPG in each of quartile groups were from −9.35 to −4.76, from −4.73 to −3.72, from −3.71 to −2.58, and from −2.57 to 0.40. ^d^ The ranges were from −6.79 to −3.44, from −3.41 to −2.27, from −2.26 to −1.22, and from −1.22 to 1.58. Adjusted ORs in female were 0.47 (0.22, 0.98), 0.50 (0.23, 1.06), and 0.32 (0.13, 0.72). Adjusted ORs in male were 0.44 (0.21, 0.92), 0.38 (0.18, 0.80), and 0.25 (0.11, 0.56). In each gender, the group with higher DPG had the lower adjusted OR for nocturia

In the stratified analysis by gender, the crude OR for nocturia was more likely to be lower along with higher daytime DPG quartile groups in each gender (*P* for trend < 0.001). In female participants, the significant trend was independent of potential confounders (> 70 years of age, BMI of ≥ 25 kg/m^2^, current smoking, and ethanol intake of ≥ 30 g/day) (*P* for trend = 0.008) (Fig. 2). In male participants, the trend stayed significant after adjusting for benign prostatic hyperplasia (*P* for trend < 0.001) (Fig. 2). Daytime DPG was not significantly associated with total nighttime urine volume (*P* = 0.385) and nighttime single-voided urine volume (*P* = 0.084). Based on the OR for nocturia in Q1–Q3 compared with Q4 in female participants (adjusted OR = 2.35; 95% CI: 1.16–4.75; *P* = 0.017) and male participants (adjusted OR = 2.09; 95% CI: 1.10–3.98; *P* = 0.025), the population-attributable fractions were 42% (95% CI: 7–77; *P* = 0.017) in female participants and 45% (95% CI: 15–74; *P* = 0.003) in male participants.

## Discussion

To the best of our knowledge, this study first showed the association between DPG and nocturia. Results showed that a 1 °C increase in daytime DPG was significantly associated with 20% lower odds of nocturia independent of potential confounders.

It is important to keep in mind that nocturia can affect nighttime DPG through the act of going to the bathroom. When we compared daytime and nighttime DPG in the above results, we found the latter was strongly associated with nocturia. In contrast, reverse causation is less likely for daytime DPG because of the temporal sequence of the events.

In our study, nocturia was more common in men, and the same was true of the previous studies [25]. The multiple factors are involved while influencing one another, including lower urinary tract obstruction caused by enlarged prostate, reduced bladder capacity, increased urine volume resulting from hormonal changes, and comorbid conditions such as heart failure [26].

Through evaluating DPG in the living environment in a large sample, we investigated two unresolved issues in previous studies, including the association between skin temperature and nocturia under real-life conditions and the effect of potential confounders. Epidemiological studies showed that nocturia was associated with a lower outdoor [15] and indoor [14] temperature. According to previous studies on rats, rapid cooling of environmental temperature [11] and spraying menthol on skin [12] caused frequent urination. Urinary volume increased with colder ambient temperature in experimental studies on 6 youngsters [27] and 10 combat swimmers [28].

Cold-induced diuresis may partly be involved in the association observed in this study. Cold-induced peripheral vasoconstriction increases central blood volume and arterial blood pressure, which lead to the stimulation of volume and pressure receptors and subsequent neuroendocrine responses including increased atrial natriuretic peptide, decreased renin-aldosterone activity, and decreased vasopressin. These changes may increase urine production. In addition, mechanisms other than increased urine production, such as impaired bladder storage function related to cold exposure, may also be involved [17]. Importantly, the present findings should not be interpreted as indicating that participants with nocturia were exposed to stronger absolute cold at night. Distal skin temperature normally rises at night as part of sleep-related thermoregulation. Therefore, the key finding is not the absolute day–night difference in skin temperature, but the relative between-group difference in DPG. In this context, lower DPG in participants with nocturia may simply reflect relative peripheral vasoconstriction or reduced heat dissipation under real-life conditions. Such a thermoregulatory state may contribute to nocturia through multiple pathways.

The mechanisms of nocturia induced by lower daytime DPG are not completely understood. However, this might be explained by increased urine production at night and lower bladder capacity. Low daytime DPG may associate with cold-related higher venous resistance, which causes fluid retention in the distal region [29] during the daytime. It may cause polyuria during the night. According to a rat experiment [11, 13], cold exposure activated transient receptor potential melastatin 8 and caused detrusor overactivity via C-fiber sensory nerves and α1-adrenergic receptors. Nitric oxide (NO) can work as a neurotransmitter in the urothelium. Cold exposure inhibits NO synthase [30], which is associated with urodynamic parameters in the human subjects with bladder outlet obstruction [31]. It is known that NO is associated with nocturia through both nocturnal polyuria and impaired bladder capacity. Reduced NO bioavailability has also been implicated in nocturnal polyuria, including salt-related mechanisms in experimental models [32]. Decreased NO production leads to detrusor overactivity through hypersensitivity to muscarinic stimulation and reduced relaxation mediated by β3-adrenoceptor [33]. Increased NO in the bladder can suppress bladder overactivity by inhibiting afferent activity via Aδ-fiber and C-fiber bladder afferent pathway [34]. Our previous study showed that higher serum levels of asymmetric dimethylarginine, an endogenous inhibitor of NO synthase, were associated with higher prevalence of nocturia in the epidemiological study [35]. This result was consistent with those of the above experimental study.

Based on the results of this study, it was possible that individual difference of endogenous factors such as NO were involved in nocturia. In model 4, it seemed that the ambient temperature did not influence the OR for nocturia through lower DPG. In this study, both participants with and without nocturia were exposed to similar ambient temperature. However, a difference was observed in DPG. The difference of DPG might be derived from other factors rather than ambient temperature.

In our data, DPG was not significantly associated with nighttime urine volume: total volume (*P* = 0.385) and single-voided volume (*P* = 0.084). This suggests that these factors alone may not account for observed difference in nocturia. Therefore, it is plausible to consider that the net effect of these factors, along with other potential variables, could contribute the significant difference in nocturia. Nighttime single-voided urine volume is associated with functional bladder storage capacity. And nighttime total urine volume is associated with polyuria. The model 6 and 7 were unable to clearly demonstrate that increased urine production or decreased functional bladder capacity was the mechanism by which lower DPG led to nocturia. In Table 2, participants with nocturia had higher abdominal skin temperature. DPG has been sometimes used as an indicator for estimating blood flow in distal skin regions and distal heat loss. It is possible that higher abdominal skin temperature may be just a result of inadequate heat loss, which can indicate peripheral vasodilation of individuals with nocturia are not functioning.

Based on the population-attributable fraction in this study, nocturia was associated with a lower daytime DPG in > 40% of cases. This result suggests that DPG can be a potential target for intervention to prevent and relieve nocturia. Although indoor temperature can be also a target [14], directly controlling skin temperature could be superior. According to previous studies, DPG increased by bathing or warming eye mask [36, 37].

The current study had several limitations. The skin temperature measurements obtained using the iButton device may be subject to measurement error due to insulation of the device and the covering material at the measurement sites [38]. However, such error is likely to be largely non-differential between participants with and without nocturia. Although a correction equation has been proposed [21], to our knowledge, no widely used correction equation exists; therefore, we did not apply any correction. When we calculated room temperature, we did not use bathroom temperature and water temperature in the bathtub. This study was conducted in a real-life setting, where the participants’ clothing changed frequently. Accordingly, we determined that assessing the type and amount of clothing, which may influence skin temperature, was quite difficult. We did not have data about consumption of soft drinks including coffee or tea. As we did not perform imaging tests, we could not assess the abdominal fat distribution. However, we used BMI as an indicator for assessing obesity. We did not collect information about daytime urination, residual urine volume, and prostate size. The current study was based on the measurements of exposure and outcomes within a short period. In the recruitment of participants, we did not conduct random sampling. The indoor temperatures measured in this study were similar to those reported in the nationwide survey in Japan [39]. Because the findings of this study are based on measurements from a single region in Japan, further research is needed to generalize the results to areas with different climates and cultural contexts. This study is a cross-sectional study and did not allow us to draw any conclusions regarding a causal relationship between DPG and nocturia. Finally, we did not take advantage of the characteristic of time-series data and mainly used the daytime and nighttime average skin temperature.

## Conclusions

A higher daytime distal–proximal skin temperature gradient in the living environment was independently associated with lower odds of nocturia in older adults. To the best of our knowledge, this study first showed the association between skin temperature in the living environment and nocturia. Apart from ambient temperature, DPG might be involved in nocturia. And DPG could be a potential target for intervention to prevent or relieve nocturia.

## Data Availability

Data cannot be shared for privacy or ethical reasons.

## Abbreviations

DPG: Distal-proximal skin temperature gradient
SD: Standard deviation
BMI: Body mass index
eGFR: Estimated glomerular filtration rate
BP: Blood pressure
OR: Odds ratio
NO: Nitric oxide
